# MoodMover: Development and usability testing of an mHealth physical activity intervention for depression

**DOI:** 10.1177/20552076251317756

**Published:** 2025-02-03

**Authors:** Yiling Tang, Madelaine Gierc, Henry La, Juehee Kim, Sam Liu, Raymond W Lam, Eli Puterman, Guy Faulkner

**Affiliations:** 1 School of Kinesiology, 8166 University of British Columbia, Vancouver, BC, Canada; 2 School of Exercise Science, Physical and Health Education, 8205 University of Victoria, Victoria, BC, Canada; 3 Department of Psychiatry, University of British Columbia, Vancouver, BC, Canada

**Keywords:** Physical activity, depression, mental health, mHealth, behaviour change, eHealth

## Abstract

**Background:**

Physical activity (PA) is recognized as a modifiable lifestyle factor for managing depression. An application(app)-based intervention to promote PA among individuals with depression may be a viable alternative or adjunct to conventional treatments offering increased accessibility.

**Objective:**

This paper describes the early stages of the development process of MoodMover, a 9-week app-based intervention designed to promote PA for people with depression, including its usability testing.

**Methods:**

Development of MoodMover followed the initial stages of the Integrate, Design, Assess, and Share (IDEAS) framework. The development process included (1) identifying intervention needs and planning; (2) intervention development; and (3) usability testing and refinement. Usability testing employed a mixed-methods formative approach via virtual semi-structured interviews involving goal-oriented tasks and administration of the mHealth App Usability Questionnaire (MAUQ).

**Results:**

Drawing on formative research, a multidisciplinary research team developed the intervention, guided by the Multi-Process Action Control framework. Nine participants engaged in the usability testing with the MoodMover prototypes receiving an average MAUQ score of 5.79 (SD = 1.04), indicating good to high usability. Necessary modifications were made based on end-users' feedback.

**Conclusions:**

The development of MoodMover, the first theoretically informed app-based PA intervention for individuals with depression, may provide another treatment option, which has wide reach. The comprehensive usability testing indicated interest in the app and strong perceptions of usability enabling a user-centered approach to refine the app to better align with end-users' preferences and needs. Testing the feasibility and preliminary efficacy of the refined MoodMover is now recommended.

## Background

Physical activity (PA) is recognized as a modifiable lifestyle behaviour with a positive impact on the prevention and management of depression.^1,2^ A recent overview of systematic reviews on the effectiveness of PA interventions demonstrated a medium effect size (median SMD: −0.66) in reducing depressive symptoms based on 14 systematic reviews comprising 273 randomized controlled trials (RCTs) among patients diagnosed with depression.^3^ In Canada^4^ and many other nations, such as the United Kingdom,^5^ exercise – identified as planned and purposeful PA – is advocated as a standalone treatment for mild-to-moderate depressive episodes. However, the global prevalence of physical inactivity (i.e., not meeting PA guidelines) among people with depression remains a significant concern.^6^ A previous meta-analysis reported that individuals with severe mental illness were 50% less likely to be physically active than healthy participants.^6^

Behavioural change interventions that promote habitual PA hold promise particularly for those who may not be interested in, or able to access, structured exercise interventions. However, very few behavioural interventions with a focus on PA promotion have been available for people with depression.^7^ Machaczek et al.^8^ found that three out of five behavioural interventions led to an increase in PA uptake among people with depression, showing improvements in direct measures of PA^9,10^ and indirect measures of PA through muscular endurance and aerobic fitness.^11^ Wong et al.^12^ identified five RCTs of multicomponent lifestyle interventions that combined a PA component and other lifestyle factors among people with major depressive disorder or depressive symptoms, noting significant positive effects on reducing depressive symptoms. Although these interventions have shown the potential to increase PA and reduce depressive symptoms, both Machaczek et al.^8^ and Wong et al.^12^ emphasized the need for interventions with greater accessibility.

Mobile applications (apps) are increasingly being utilized to implement interventions aimed at behaviour change because of their wide reach and ubiquity.^13,14^ In Canada, national data from 2020 showed that 84.4% of Canadians aged 15 and over owned a smartphone, and this ownership rate continues to rise annually.^15^ Moreover, over three-quarters of individuals with depressive or anxiety symptoms reported having health apps on their devices, with exercise and mental health apps ranking among the most common categories.^16^ Collectively, these findings point to the potential feasibility of app-based lifestyle modifications in PA as an option for treating depression on a large scale.

However, our previous systematic review highlighted the deficit of PA behavioural apps for depression.^17^ As of November 2021, only one study by Guo et al.^18^ evaluated such an intervention reporting effectiveness in reducing depressive symptoms, but not in promoting PA when compared to a waitlist/usual care control group. Their PA component, lacking both a theoretical basis and systematic development, was less amenable to rigorous evaluation and improvement. Additionally, low user engagement – a prevalent issue among apps for this population^19–21^ – may contribute to its limited impact. This review underscored the need for well-designed apps that improve user engagement to effectively increase PA in people with depression.

**Figure 1.**
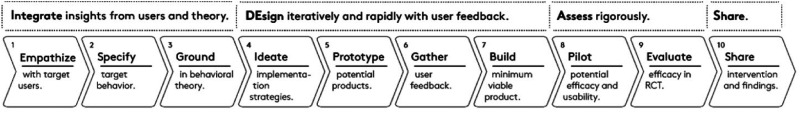
The IDEAS framework.

One approach to enhance the efficacy and sustainability of behavioural interventions is to develop such interventions following a rigorous intervention development process.^22^ This study selected the Integrate, Design, Assess, and Share (IDEAS) framework^23^ that was specifically proposed for facilitating the development process of digital behavioural interventions and fostering app creativity. Overall, this paper describes the first two stages – Integrate and Design – of the development process for the app-based intervention, MoodMover, which was designed to increase PA among individuals with depression, following the IDEAS framework (Figure 1).

## Methods and results

The development process consisted of three main phases: (1) identifying intervention needs and planning (Steps 1 and 2 of IDEAS); (2) intervention development (Steps 3–5 of IDEAS); and (3) usability testing and refinement (Steps 6 and 7 of IDEAS).

### Phase I: Identifying intervention needs and planning

#### Intervention needs

During this phase, we first conducted a systematic review of internet-based self-guided interventions aimed at increasing PA for depression to determine the intervention needs and potential intervention components and strategies.^17^ This review identified that few mobile apps designed to increase PA specifically target individuals with depression, highlighting the need for well-designed apps with enhanced engagement and efficacy. In response, we followed the IDEAS framework to design MoodMover. A multidisciplinary team that consists of professionals in the field of depression and PA was involved in the entire development processes.

##### M-PAC framework

Like other behavioural development frameworks, the IDEAS framework also emphasizes the importance of incorporating behaviour change theories into the development processes for identifying influential factors and potential explanations for the failure of interventions.^23–26^ Instead of traditional social cognitive theories, such as the theory of planned behaviour and the social cognitive theory, the multidisciplinary team selected the Multi-Process Action Control (M-PAC) framework^27,28^ with an acknowledgement of the intention–behaviour gap, where merely having an intention to engage in PA does not always lead to behaviour change.^29^

In the M-PAC framework, intention is viewed as a decisional construct, with core determinants that include reflective processes (i.e., instrumental attitude, affective attitude, perceived capability, and perceived opportunity). Among these, two ongoing reflective constructs (perceived opportunity and affective attitude) along with regulatory processes (e.g., action/coping planning) collectively facilitate the intention–behaviour transition (action control). Furthermore, the M-PAC framework emphasizes the importance of two reflexive processes, namely, habit and identity, in sustaining effective action control. The M-PAC has been applied to understand and promote PA in various populations^30–34^ and has been deemed suitable for informing PA promotion interventions among people with poor mental health.^35^ Moreover, the M-PAC framework includes a set of theory-driven behaviour change techniques (BCTs),^36^ which guide intervention content development.

#### Target behaviour

We conducted an exploratory literature scan to determine the intervention target behaviour, i.e., what ‘dose’ of PA is needed for a clinically meaningful reduction in depressive symptoms. Various PA guidelines encourage the general population to engage in at least 150 min (30 min/day × 5 days) of moderate PA per week for substantial health benefits (e.g., Ross et al.^37^ and Bull et al.).^38^ Walking is the primary form of PA behaviour targeted by MoodMover due to its accessibility, countability, and ability to be incorporated into the daily lives of all people without severe physical disabilities. A meta-analysis by Robertson et al.^39^ suggested that walking exhibits a similar effect size for treating depression or depressive symptoms compared to other types of exercise.

A 30-min session of moderate-intensity walking is estimated to accumulate around 3000–4000 steps.^40–42^ As such, an increase of 3000 daily steps was deemed a clinically relevant behavioural goal for MoodMover. In addition, a commentary by Otto et al.^43^ on exercise for depression suggested adding steps to individuals' baseline PA levels gradually. Similarly, previous web-based PA interventions proposed and applied ramped goals of 1000 extra steps above baseline every 2 weeks until achieving a total increment of 3000 steps.^44,45^ Despite less than half of the participants achieving the ultimate goal, both studies indicated significant increases in step counts at post-intervention.

**Figure 2.**
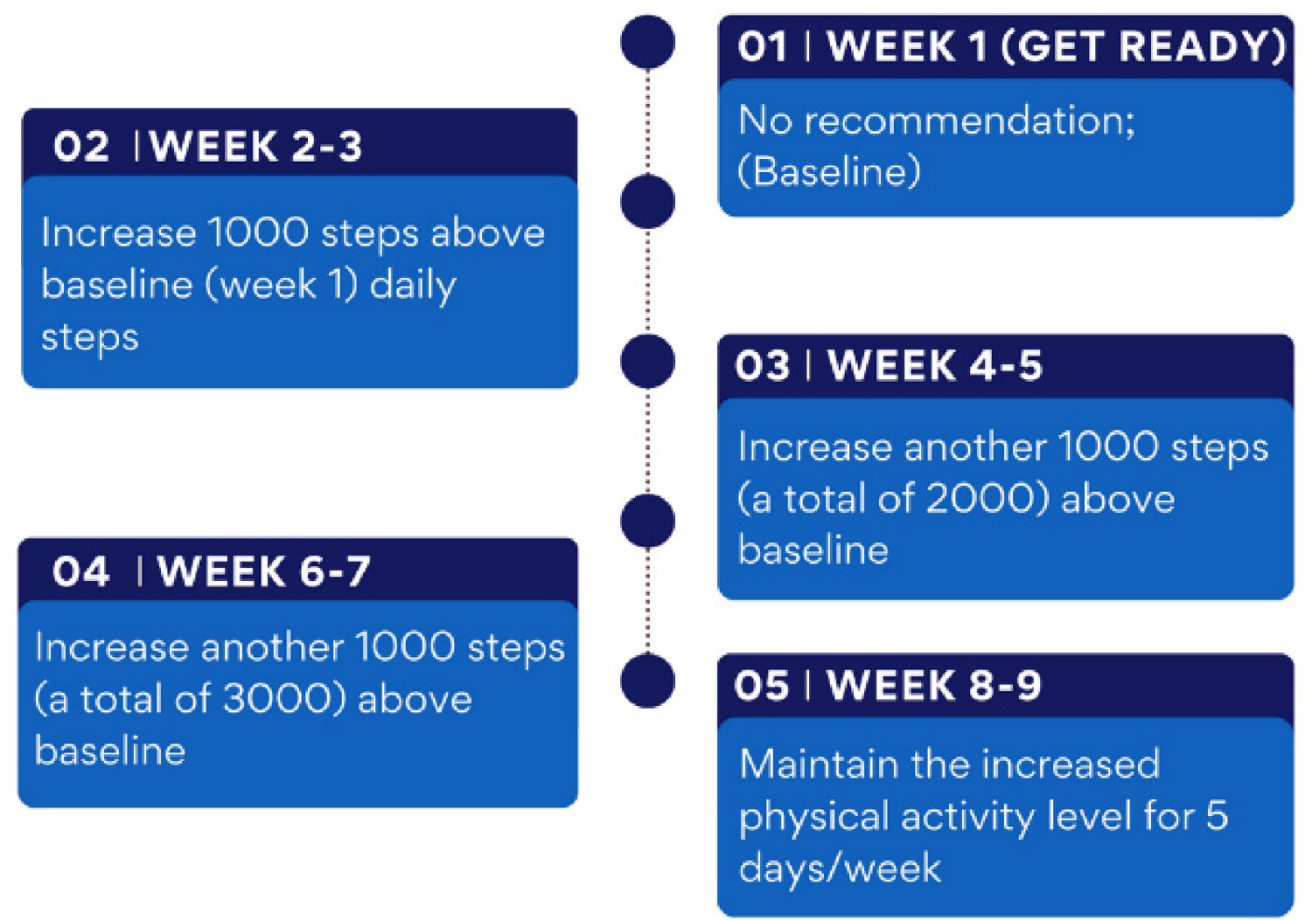
Graded goal recommendations of MoodMover.

Given these, MoodMover adopted a similar graded approach by recommending participants to set their goals as increasing 1000 steps/day above baseline at Week 2, followed by adding an additional 1000 daily steps every 2 weeks until meeting a total increase of 3000 steps/day (see Figure 2). This aligns with the findings of Sporrel et al.,^46^ suggesting that assigning goals to users and using adaptively tailored goals may be more effective than user-set or generic goals. Moreover, MoodMover encourages end-users to gradually increase their daily goal achievement to 5 days/week, aligning with the potential importance of frequency in cultivating PA habits.^43^ This goal is likely to be both realistic and effective for individuals with depression.

### Phase II: Intervention development

During this phase, the multidisciplinary team discussed content modifications, brainstormed potential app features and modes of delivery, and developed prototypes. This development process was highly iterative, being repeated until the research team reached consensus that the prototype was ready for usability testing.

#### Intervention content

The content of MoodMover was adapted from an existing web-based, 10-lesson M-PAC intervention designed to promote PA among young adults with low mood and/or depression engaged in community primary clinical care.^47^ This program was selected for adaption due to its good initial acceptability among a similar population of individuals with poor mental health and its alignment with the M-PAC framework. The adaption of the content was guided by previous literature with consultations with the professionals in our multidisciplinary team.

A recent study by Tang et al.^35^ underscored the potential significance of certain M-PAC constructs, namely, capability, affective attitudes, intentions, and regulatory processes, in promoting PA among individuals with poor mental health. Conversely, perceived opportunity and instrumental attitudes were less pronounced in this population. In response, while the modified content retains all the constructs of the M-PAC framework, it places more emphasis on perceived capability, affective attitudes, regulatory processes, and reflexive processes but less emphasis on perceived opportunity and instrumental attitudes. This focus on affective attitudes is consistent with evidence from other behaviour change frameworks, suggesting that emotion domains may be a crucial aspect that has been overlooked in previous interventions targeting changes in PA behaviour among individuals with depression.^7^ All theoretical constructs have been mapped to BCTs. Table 1 presents an overview of MoodMover's content of major lessons, targeted M-PAC constructs, and corresponding BCTs; a full version with complementary lessons is shown in Supplementary Appendix 1.

**Table 1.** Outline of the content of MoodMover's major lessons and the corresponding M-PAC construct(s) and behaviour change technique(s).

Modules	Major topics covered	M-PAC construct(s) targeted	Behaviour change technique(s) used
1. Feeling better through daily activity	Exercise as a treatment option for depression Mood and physical activity interaction Physical benefits of exercise Goal-setting	Affective attitudes, instrumental attitudes; behavioural regulation	Information about health consequences (5.1)^a^ Information about emotional consequences (5.6)^a^ Behavioural practice/rehearsal (8.1)^a^ Goal setting (behaviour) (1.1)^a^ Self-monitoring of behaviour (2.3)^a^ Credible source (9.1)^a^
2. Making physical activity enjoyable	Introducing affect The importance of enjoying physical activity Strategies and activities to increase the enjoyment of physical activity	Affective attitudes; behavioural regulation	Information about emotional consequences (5.6)^a^ Behavioural practice/rehearsal (8.1)^a^ Self-monitoring of behaviour (2.3)^a^
3. Building your self-confidence	Self-efficacy How to increase self-efficacy Exercise experiences shared by peers	Perceived capability; behavioural regulation	Goal setting (behaviour) (1.1)^a^ Self-monitoring of behaviour (2.3)^a^ Information about others' approval (6.3)^a^ Behavioural practice/rehearsal (8.1)^a^
4. Building your physical activity opportunity	The influence of environment on behaviour How to build environment for physical activity Brainstorm physical activity opportunities Grab and go activities	Perceived opportunity	Goal setting (behaviour) (1.1)^a^ Self-monitoring of behaviour (2.3)^a^ Prompts/cues (7.1)^a^ Behavioural practice/rehearsal (8.1)^a^ Restructuring the physical environment (12.1)^a^ Adding objects to the environment (12.5)^a^
5. Developing self-regulatory skills	Action planning Coping planning	Behavioural regulation	Goal setting of behaviour (1.1)^a^ Problem solving (1.2)^a^ Action planning (1.4)^a^ Self-monitoring of behaviour (2.3)^a^ Behavioural practice/rehearsal (8.1)^a^
6. Drawing on social support	Introducing social support Building your social support Thinking strategies for exercise Positive self-talk	Perceived opportunity; behavioural regulation	Goal setting (behaviour) (1.1)^a^ Self-monitoring of behaviour (2.3)^a^ Social support (practical) (3.2)^a^ Social support (emotional) (3.3)^a^ Behavioural practice/rehearsal (8.1)^a^ Self-talk (15.4)^a^
7. Forming an exercise habit	Introducing habit Relating habit to physical activity How to form a habit	Habit	Goal setting (behaviour) (1.1)^a^ Self-monitoring of behaviour (2.3)^a^ Behavioural practice/rehearsal (8.1)^a^ Habit formation (8.3)^a^
8. Building your exercise identity	Introducing WHO guidelines Introducing exercise identity Ways to increase exercise identity	Identity	Goal setting (behaviour) (1.1)^a^ Self-monitoring of behaviour (2.3)^a^ Behavioural practice/rehearsal (8.1)^a^ Incompatible beliefs (13.3)^a^ Valued self-identity (13.4)^a^

*Note*. M-PAC: Multi-Process Action Control; WHO: World Health Organization.

^a^ The numbers in the brackets refer to the behaviour change techniques in the ‘BCT taxonomy v1’ by Michie et al.^36^

Despite the original content of the web-based intervention being evaluated in collaboration with end-users and researchers and incorporating various BCTs,^47^ we reassessed the alignment of targeted theoretical constructs with the taxonomy of BCTs^36^ to identify additional intervention components that may be effective and preferred by end-users.

Overall, the revised program consists of eight major lessons accompanied by eight weekly complementary lessons. With an additional run-in period of 1 week (Week 1) for introducing the program and obtaining baseline step data, MoodMover results in a 9-week intervention. The major lessons generally follow the order of the three processes in the M-PAC framework, beginning with intention-formation (Lessons 1–4; 6), followed by action control adoption (Lessons 1 and 2; 4–6), and concluding with action control maintenance (Lessons 7 and 8). It is worth noting that constructs for intention–behaviour transition (i.e., perceived opportunity, affective attitudes, and regulatory processes) were emphasized early and integrated throughout the program, with the assumption that most individuals who volunteered to participate in a PA behavioural intervention already had intentions to be physically active.^48^ Several behavioural strategies, such as positive self-talk after exercising and increasing pleasant activities recommended by Otto and Smits,^49^ were incorporated. To match the temporal dynamics of symptom severity, especially for energy and motivation,^50,51^ MoodMover added information about the fluctuating nature of depression and implemented strategies to emphasize engaging in PA when feeling more energetic and less fatigued.

#### Prototype development

The research team iteratively developed multiple prototypes on Pathverse, continuously refining the app's content, features, navigation, layout, and aesthetic design. Pathverse is a ‘no code’ mHealth intervention development platform^52^ (see https://pathverse.ca/en/), which aligns with the M-PAC framework and enables the creation of tailored behavioural interventions. It supports rapid prototyping, allowing the customization of app content and features through a web portal. Three independent researchers (GF, MF, and MG) assessed the content and the design of the app and provided feedback for making necessary modifications. Each modification of the app content and design was reviewed and approved by at least three team members. Any discrepancies were addressed through discussions within the research team to reach a consensus. A computer programmer (HL) from Pathverse was engaged in this iterative process to provide technical support.

During this process, a few major modifications were implemented. For example, to further enhance engagement and increase PA, financial incentives (BCT: ‘10.1 Material incentive (behaviour)’) were incorporated into the gamification feature that Pathverse has enabled. Gamification features are associated with increased engagement in PA apps and increased PA levels.^53^ MoodMover incentivizes lesson completion, allowing participants to earn 20 points for each major lesson and 10 points for each complementary lesson. Every 60 points earned are equal to a CAD $5 e-gift card.

In addition, inspired by Webb et al.^54^ to enhance practical apps, two modes of delivery – ‘automated tailored feedback’ and ‘enriched information environment’ – associated with higher effects on behaviour change were considered. Unfortunately, due to limited resources and time constraints, ‘automated tailored feedback’ was not incorporated. Instead, the prototype of MoodMover included an enriched information environment (i.e., images, videos, and short podcasts), along with automated follow-up messages (e.g., reminders/notifications), and peer-to-peer access (i.e., community forum). Furthermore, the prototype incorporated a mood-tracking feature by adapting the existing exercise logging feature, potentially reinforcing the sense of connection between PA and mood enhancement.

**Figure 3.**
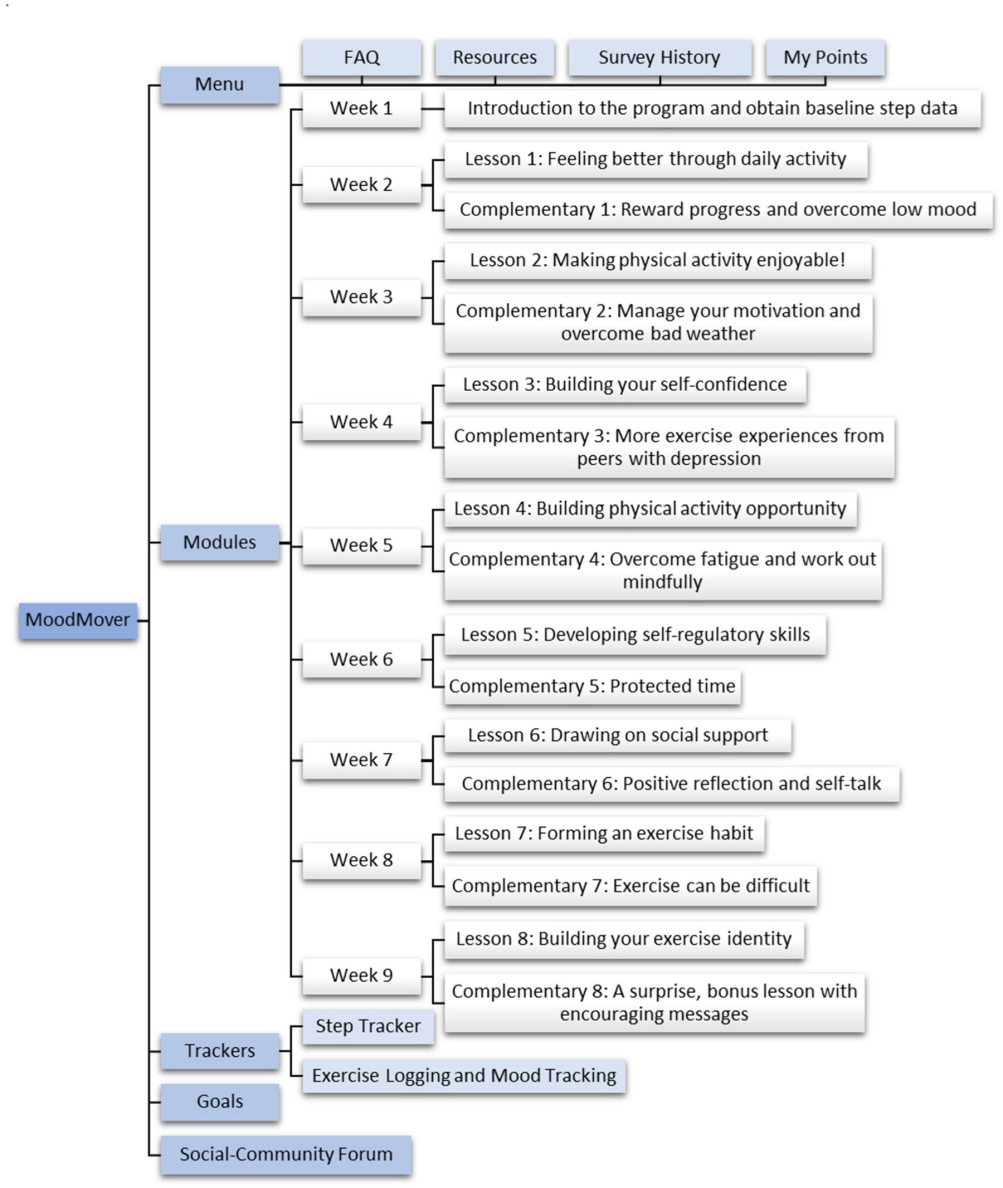
A workflow of the refined prototype of MoodMover.

**Figure 4.**
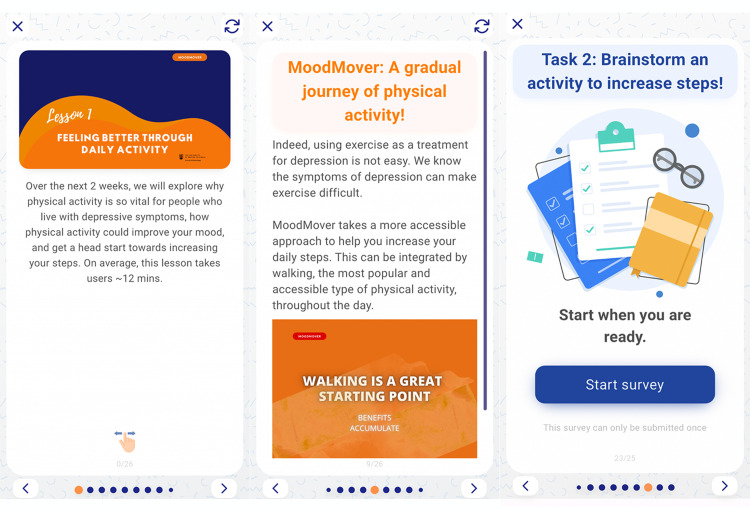
Screenshots of a major lesson.

Overall, the refined prototype contains various features (e.g., step tracker and community forum) and lessons with content framed within the M-PAC framework (one major/week and one complementary/week). Figure 3 illustrates a workflow of the refined prototype of MoodMover. Each lesson, except for Week 1 introduction module, includes educational content presented on lesson cards in a variety of formats (e.g., texts, images, GIFs, and podcasts), along with surveys and/or quizzes. Figure 4 displays screenshots of a major lesson as an example.

### Phase III: Usability test

After the research team was satisfied with the modified intervention package, a usability test of the MoodMover prototype was conducted to receive user feedback. This phase aims to explore users' general interest in the app and their willingness to integrate it into their regular routines. It also sought to identify any potential barriers to consistent usage, gather suggestions for improvement, and capture unexpected innovative ideas or opportunities that might arise. To comprehensively analyse MoodMover from end-users' perspectives, we replicated a prior usability study of a Pathverse app^55^ in employing a formative mixed methods approach. Ethics approval to conduct this study was received by the Research Ethics Board at the University of British Columbia (#H24-00047). Supplementary Appendix 2 presents the COREQ (COnsolidated criteria for REporting Qualitative research) checklist.

Participants were recruited through REACH BC (https://reachbc.ca/), an initiative in British Columbia, Canada, that facilitates participant recruitment for health research. Potential participants who expressed interest were contacted by one researcher (YT) via email and invited to complete a screening questionnaire (Supplementary Appendix 3) online to confirm their eligibility. All eligible patients received further information about the study via email and completed a demographic survey (Supplementary Appendix 4; e.g., gender) after confirming their participation prior to the meeting. All participants provided verbal consent at the beginning of the Zoom meeting on the recording. Written informed consent was waived by the Research Ethics Board. Inclusion criteria for participants include (1) outpatients aged 18–64 years who self-report a current diagnosis of major depressive disorder, irrespective of its severity, and/or at least mild depressive symptoms, as indicated by a score of 5 and above (mild: 5–9, moderate: 10–19, and severe: 20–27) on the nine-item Patient Health Questionnaire (PHQ-9)^56^; (2) have a valid email address; (3) possess an iPhone or Android smartphone with internet access to download and use the app; and (4) able to read and write in English. Patients with the following self-reported conditions were excluded: physical disability preventing exercise, active psychosis or mania, active suicidal ideation, severe cognitive impairment, and current pregnancy. Participants who reported 90 min or more of at least moderate-intensity PA per week were also considered ineligible. Participants were compensated with a CAD $20 Amazon e-gift card for their time.

#### Procedures and measurements

Participants were asked to sign up on Pathverse to access MoodMover using the same email through which they had been contacted by the researcher. The virtual interviews lasted approximately 60 min and were conducted over participants' smartphones by the first author who has received graduate training in qualitative research. During the meeting, participants first partook in a series of goal-oriented tasks while sharing their phone screens, aimed at obtaining user feedback and identifying potential navigation issues within specific areas of the prototype. The tasks included (1) logging onto the app; (2) completing the introduction module; (3) syncing steps from either Google Fit (Android) or Health (iOS); (4) completing the first major lesson; (5) setting a personal step goal; (6) logging one exercise session; (7) completing one complementary module; (8) scanning the fifth lesson (Self-regulatory skills) and creating an action plan after watching the tutorial embedded; and (9) scanning the sixth lesson (Social support) and sharing their experience in the community forum. Participants were encouraged to ‘think-aloud’, sharing their ongoing thought processes and any challenges they encountered while using the program throughout these tasks. A separate research assistant (JK) served as an observer, responsible for documenting any issues encountered by participants during the tasks. Both the transcriptions of the ‘think-aloud’ sessions and the field notes were integral to the data analysis process. After completing the goal-oriented tasks, a semi-structured interview followed aiming at gathering open-ended feedback and obtaining deeper insights. Interview questions (e.g., ‘what did you like best about the MoodMover app?’; see details in Supplementary Appendix 5) were mainly derived from the previous usability testing study of a Pathverse app.^55^

Last, participants completed a usability evaluation tool (Supplementary Appendix 6) revised from the patient version of the mHealth App Usability Questionnaire (MAUQ)^57^ for standalone apps. MAUQ has been considered the gold-standard reference for usability analysis of mHealth apps.^58^ The original MAUQ is a new, 18-item validated usability questionnaire, assessing three domains of usability guided by the International Organization for Standardization^59^ definition of usability: ease of use (MAUQ_E; five items), interface and satisfaction (MAUQ_I; seven items), and usefulness (MAUQ_U; six items). Given that MoodMover does not provide healthcare services, its usefulness was assessed with a single item: ‘The app would be useful for my mental health and well-being’. Participants were asked to rate each item on a 7-point Likert scale ranging from 1 (strongly disagree) to 7 (strongly agree). A higher total score and averages of each domain indicate better app usability. In its validation study, the MAUQ demonstrated strong criterion validity, as indicated by high positive correlation coefficients with both the Post-Study System Usability Questionnaire and its subscales, as well as with the System Usability Scale, with correlations ranging from *r* = .72–.86.^57^ Additionally, construct validity was supported by a high positive correlation coefficient between MAUQ_I and MAUQ_E (*r* = .75).^57^ The internal consistency of the MAUQ_E (Cronbach alpha = 0.847) and MAUQ_I subscales (Cronbach alpha = 0.908) is high.^57^ Since no specific threshold of the MAUQ has been established to indicate good usability, we set a cut-off score of ≥5 for each domain to indicate acceptable usability, based on previous mHealth app usability studies.^60,61^

#### Data analysis

Quantitative data were analysed using Microsoft Excel to describe participant demographics and summarize the MAUQ results descriptively. The interviews and think-aloud recordings were independently transcribed verbatim using Otter (www.otter.ai) and analysed in Microsoft Excel by two researchers (YL and JK). Any discrepancies were resolved through discussions to ensure the consistency of analyses. Qualitative content analysis approach^62^ was employed for its ability to provide consistent content categories of interest and flexibility in identifying emerging patterns. The data analysis began after the first usability session was conducted to ensure that issues identified in early sessions can inform later sessions using constant comparative analyses. Additionally, some of the issues identified (e.g., functional bugs) and suggestions raised by previous participants were used to inform ongoing modifications, which were then evaluated by the subsequent participants. Using deductive content analysis, the collected data were coded to correspond to three broad categories: app design (e.g., layout, navigation and aesthetics), content (e.g., educational material) and features (e.g., step tracker), and ideas for improvement. These data were cross-referenced with notes taken by the researcher (JK) during the think-aloud process. Subsequently, inductive content analysis was also used to further explore subcategories.

#### Results of usability test

A total of 49 individuals who expressed interest in participating through REACH BC were contacted. Of the 19 individuals who met the eligibility criteria, 12 (63.2%) were enrolled, while the remaining individuals did not respond to the invitation emails. Of these enrolled participants, two did not attend the scheduled meeting without providing a reason. One participant was excluded due to technical issues not related to Pathverse/MoodMover. The remaining nine participants (five women, three men, and one non-binary) had a mean age of 38.4 years, ranging from 24 to 53 years. Six participants self-reported a clinical diagnosis of major depressive disorder. All participants self-reported a mean of 14.1 points on the PHQ-9. Of these, three scored within the mild threshold, while four were categorized as moderate, and two as severe. Further demographics are displayed in Supplementary Appendix 7.

Due to time constraints, only the first seven goal-oriented tasks were assigned to all participants. Task 8, scanning the fifth lesson (self-regulatory skills) and creating an action plan, was assigned to three participants. Task 9, which involved scanning the ‘Lesson 6: Social Support’ and sharing their experience in the community forum, was not assigned; instead, participants were introduced to the ‘social community forum’ feature and asked to provide feedback.

##### Quantitative results: MAUQ

Table 2 presents the results of the adapted MAUQ. The prototypes of MoodMover received an average MAUQ score of 5.79 (SD = 1.04), ranging from 5.08 to 6.62 across most (8/9) participants, indicating good to high usability. When looking at the individual domains, MoodMover received an average score of 5.84 (SD = 0.88) for ‘ease of use’, with 91.1% (41/45) of the responses scoring 5 (‘somewhat agree’) or higher. In the ‘Interface and satisfaction’ domain, the average score was 5.67 (1.25), with 81.0% (51/63) of responses being at least ‘somewhat agree’. The average score for ‘usefulness’ was 6.33 (SD = 1.00), with eight (88.9%) participants scoring 6 or 7.

**Table 2.** Usability of MoodMover prototypes as assessed by the adapted MAUQ for standalone apps.

Domains and statements	Mean (SD)	Median (Min, Max)
**Ease of use**	5.84 (0.88)	6.20 (3.80, 6.60)
The app was easy to use.	5.67 (1.00)	6 (4, 7)
It was easy for me to learn to use the app.	6.00 (0.87)	6 (5, 7)
The navigation was consistent when moving between screens.	5.56 (1.13)	6 (3, 7)
The interface of the app allowed me to use all the functions (such as setting a step goal, logging a physical activity session, receiving notifications) offered by the app.	5.67 (1.87)	6 (1, 7)
Whenever I made a mistake using the app, I could recover easily and quickly.	6.33 (1.00)	7 (4, 7)
**Interface and satisfaction**	5.67 (1.25)	5.71 (2.71, 6.71)
I like the interface of the app.	5.56 (1.94)	6 (1, 7)
The information in the app was well organized, so I could easily find the information I needed.	5.44 (1.13)	6 (4, 7)
The app adequately acknowledged and provided information to let me know the progress of my action.	5.56 (0.73)	6 (4, 6)
I feel comfortable using this app in social settings.	5.44 (1.94)	6 (1, 7)
The amount of time involved in using this app has been fitting for me.	5.67 (1.66)	6 (2, 7)
I would use this app again.	6.22 (1.20)	7 (4, 7)
Overall, I am satisfied with this app.	5.78 (1.56)	6 (2, 7)
**Usefulness**		
The app would be useful for my mental health and well-being.	6.33 (1.00)	7 (4, 7)
**Overall MAUQ**	5.79 (1.04)	6 (3.38, 6.62)

*Note*. MAUQ: mHealth App Usability Questionnaire.

##### Qualitative feedback

Table 3 displays a summary of participants' responses to semi-structured interview questions with sample quotations. More detailed usability results from the think-aloud processes and semi-structured interviews are categorized into three predefined broad areas: app design, content and features, and ideas for improvement.

**Table 3.** Interview question responses.

Interview questions	Summary	Sample quotations
Q1: Do you use, or have you used, any mental health/PA apps/wearable devices? Why/why not?	Most participants reported having used PA or mental health apps before at least for short periods of time. Some participants expressed a clear need for a PA app tailored for people with depression.	It's actually surprising that no one has done this [develop an app specifically designed for depression] yet … especially since depression is such a common mental health issue. (Mood23)
Q2: What did you like best about the MoodMover app?	Gamification with incentives, exercise logging with mood tracking, and resources with YouTube workout videos were the top three mentioned features. Many participants also favoured the flexible attitude conveyed by MoodMover.	I like that the more activities you do, you accumulate points. (Mood04)
Q3: What did you like least about the app?	No major issues were reported. Some participants mentioned that the initial navigation took some time.	There's nothing that really strikes me. (Mood09)
Q4: How easy was it to navigate or find your way around the app?	Most participants explicitly commented that the app is intuitive, simple, or easy to navigate, although the initial navigation may take them some time.	I feel it was pretty easy… Like even without the instructions. (Mood42)
Q5: What did you think about the overall look of the app?	Most participants favoured the overall look of the app, except for two of three men.	Pretty nice overall look of the app. (Mood49)
Q6: What did you think about the information provided on the app?	Most participants explicitly stated that the content is very helpful, and the scripting is basic and easy to understand.	Very helpful … explained in a very simple, easy to understand way. (Mood04)
Q7: Is there anything you think the app might be missing?	Strategies. A participant mentioned providing daily suggestions for accumulating steps.	Maybe a daily suggestion of how to get more steps or something like that. (Mood05)
Q8: Would you be interested in using the upgraded MoodMover in the future to increase your PA and reduce depressive symptoms? Why/why not?	Nearly all participants showed a strong interest in using the upgraded MoodMover in the future, even for Mood02 who recorded a relatively low score on MAUQ.	Definitely. Why not? Because at the end of the day, it's all resources at one place. And again… I know that there are researchers, there are experts behind designing the app that shows you tangible benefits. (Mood02)

*Note*. MAUQ: mHealth App Usability Questionnaire; PA: physical activity.

##### App design

No participants encountered major difficulties in navigating through the app. All participants logged onto the app (Task 1) without additional instructions. Most participants experienced no issues completing the lessons (Tasks 2, 4, 7, and 8). An issue encountered exclusively by Android users was that the keyboard hid part of the textbox when entering survey responses, which has been reported to the Pathverse team. Additionally, four participants did not notice that some lesson cards were longer and required scrolling down for more information. To address this, relevant instructions were added and the content of many longer cards was divided into two or more cards as suggested. In terms of layout, three participants preferred the ‘My Points’ tab, which shows participants' accumulated points, to be displayed on the main page rather than under the ‘Menu’. Comments relating to aesthetics were positive. In particular, participants appreciated the selection of pictures and the colours.

I love your pictures. Good selection of pictures really representing lots of different people … good colors, bright colors, stimulating colors … those were all visually soothing, but inspiring. Like they're motivating colors. (Mood04)

##### Content and features

###### Content

Participants mentioned their favourable perceptions of the gradually increasing step goal recommendations and the nonjudgmental manner consistently conveyed by the app, highlighted by the flexibility it encourages in meeting step goals. Most participants deemed the recommended step goals achievable, manageable, or attainable. Some users got confused by the examples of step goals (i.e., a baseline of 2000 daily steps) provided on the goal recommendations card, which was then removed. Mood07 suggested adding the time equivalence of steps when introducing the recommended step goals. This information was added as a tip card, which many subsequent participants appreciated. In terms of content design, all participants favoured the incorporation of mixed media (e.g., images, GIFs, and podcasts) within the lessons. Some participants explicitly preferred fewer plain texts and more infographics.

###### Features

Key features were evaluated through tasks including syncing steps (Task 3), setting step goals (Task 5), logging an exercise session (Task 6), and creating an action plan (Task 8). With the intention of developing a self-help app requiring minimal human support, participants were expected to complete the tasks after reading the instructions provided in the corresponding lessons. However, many participants needed some verbal guidance, even though these were generally minimal.

##### Step tracker and exercise logging

Both tools were embedded within the ‘trackers’ tab. Most participants agreed that the instructions were straightforward and easy to understand, although some participants had questions about whether MoodMover can sync step data from their fitness trackers. This issue was resolved by adding a Q&A card that explains the app only retrieves step data from fitness trackers linked to Google Fit or Apple Health. However, many participants forgot the instructions after completing the module and needed additional verbal instructions to locate the tab.

There are instructions in the module… And then once I completed the module and want to go do what I had to do. I forgot a little bit and I had to find my way to things. (Mood05)

In response, the instructional infographics were transformed into short videos. The videos were favoured by subsequent participants over the initial infographics and were further refined based on their feedback, including a change from landscape to vertical. Regarding exercise logging, most participants particularly appreciated this feature, especially for its encouragement of logging short sessions (e.g., 5 or 10 min) and the ability to record their mood after exercising using emojis.

That's cute… It is very motivating to have the option to put the mood. (Mood23)

##### Gamification: My Points

**Figure 5.**
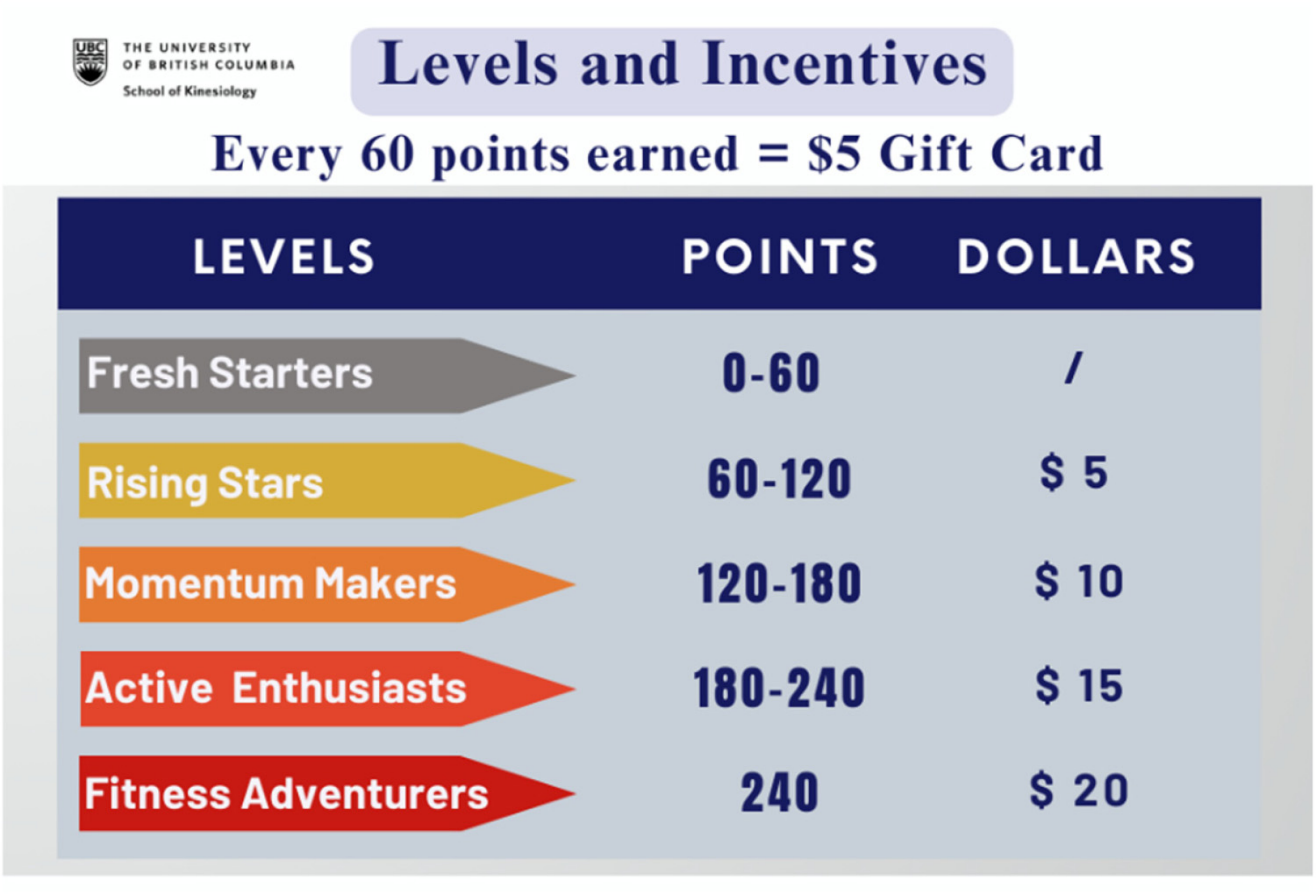
Point levels and incentives.

Figure 5 illustrates different levels of earned points and their corresponding incentives. Most participants were surprised by and excited about this feature. When participants were asked whether the amount of money corresponding to the points (i.e., every 60 points = $5) was motivating, most said they would be motivated, particularly because not much commitment was required.

It doesn't matter. It could be $1 … and I'd still be like, ‘I'm getting something’. (Mood49)

##### Goals: Action planning

**Figure 6.**
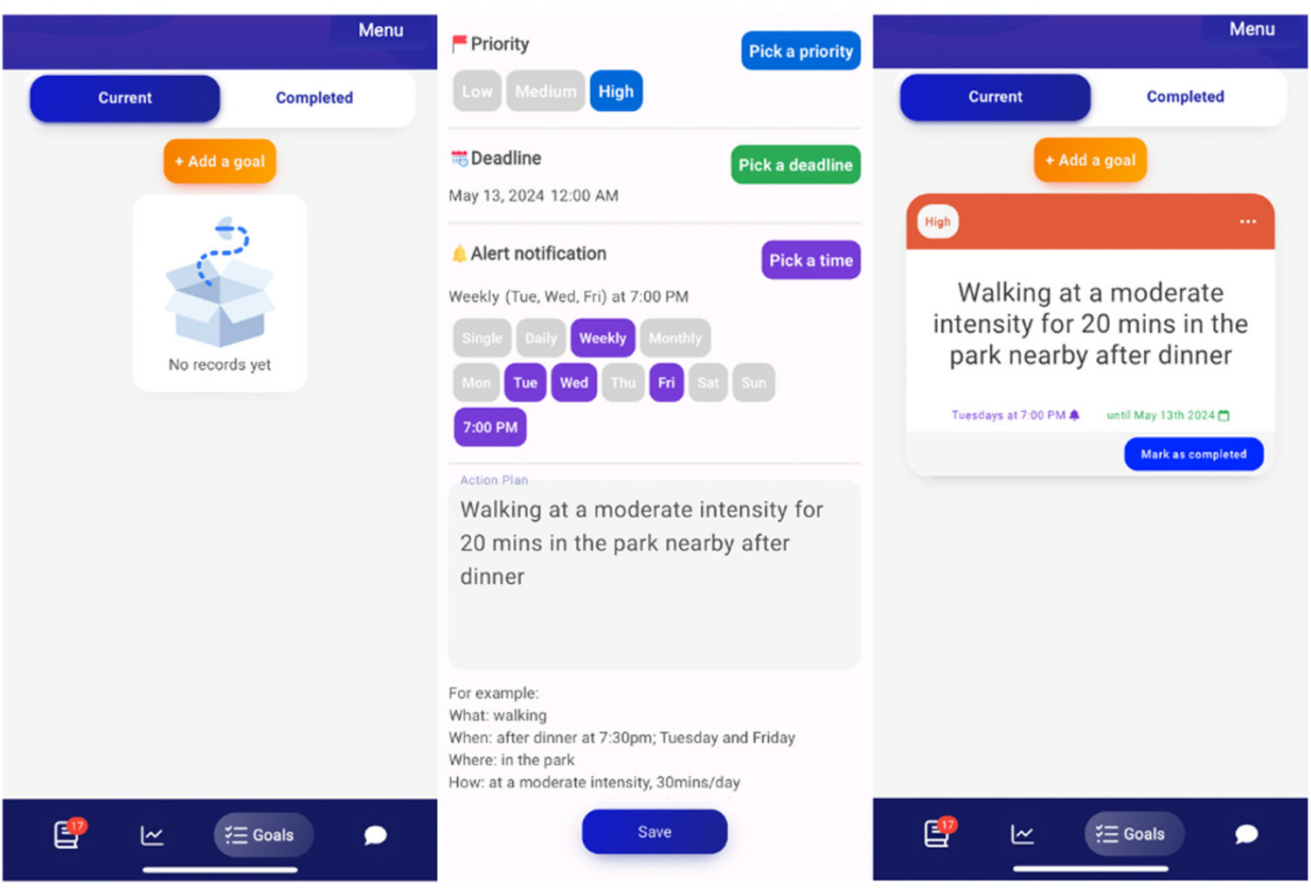
Screenshots of action planning.

Action planning was implemented using the ‘goals’ feature (Figure 6). Mood42 was excited about the ability to review a list of completed action plans, and stated, ‘I'd have a list of all of them … that's cool’. However, two issues were identified. First, the tab was labelled ‘goals’, which caused confusion with the step ‘goal’ within the step tracker. Second, the action plan example listed below the textbox was obscured when the keyboard appeared.

##### Social community forum

Only two participants showed interest in and favoured the social feature, while most participants expressed a lack of interest. After being told that the forum was completely anonymous, some participants showed increased interest.

If it's anonymous, then I might be inclined to use it, especially on a day where I'm struggling, I might just check in and see ‘is anyone else having a hard time getting out walking today?’ I know I am. It might be a way to provide some support without feeling vulnerable or exposed. So, I think that can be a really helpful tool. (Mood07)

###### Resources

Most participants particularly appreciated the curated selection of workout YouTube videos with different durations, intensity, and types. Three of them mentioned this feature as one of the best parts of the app. In particular, many participants favoured the selected walking workout videos designed to help accumulate a certain number of steps, which can align with the recommended step goals of MoodMover. More relevant YouTube videos were selected by the researcher and grouped into a separate subcategory titled ‘Videos for Accumulating Steps’.

##### Ideas for improvement

Table 4 shows a list of other ideas for improvement along with sample quotations. Minor modifications were made immediately following the interviews. Further adjustments were discussed with the Pathverse development team to enhance the usability of MoodMover.

**Table 4.** A list of additional ideas for improvement with sample quotations*.*

Ideas for improvement	Modifications or actions
Layout	
‘I think just having a date up here [lesson previews] that says coming on this day … would be super helpful.’ (Mood47)	Consulted with the Pathverse team to add scheduled release dates on the lesson previews.
Navigation	
‘I would expect this to be the main page because there's only one program here.’ (Mood49)	Consulted with the Pathverse team to set the study page as the main page for participants registered in only one study
Content	
‘Add a disclaimer on the top to remind participants even if there are many cards in one lesson. Each of them includes very limited content.’ (Mood02)	A tip card was added
Exercise logging and mood tracking	
‘People with depression is almost never happy, and the neutral is just blah. We are usually in between.’ (Mood23)	Consulted with Pathverse team to add additional mood choices between neutral and happy.
Goals	
‘I think it would be nice to have a box for each thing … instead of an action plan box. If there was a “What” box, “When” box, “Where” box.’ (Mood42)	Consulted with Pathverse team to break down for each domain
Social – Community Forum	
‘There Should Be Something Where You Can Connect To Technicians For Any Technical Problems.’ (Mood02)	Added A Topic ‘If You Need Technical Support…’ To Allow Users To Report Technical Issues Using The Social Feature

## Discussion

This study describes the development of the first app-based intervention focused on increasing PA among individuals with depression, grounded in the M-PAC framework. The study adhered to the first five steps of the IDEAS framework to iteratively develop prototypes and proceeded to the sixth and seventh steps for evaluating the usability of the prototype among end-users using a mixed-methods approach and refining the prototype. The involvement of a multidisciplinary team facilitated the app's development, bringing more divergent and creative ideas while enabling the convergence of solutions to efficiently refine the prototype.^23^ The highly iterative, user-centred development processes enhanced the usability and acceptability of the tested MoodMover prototypes by aligning with end-users' needs.

The development of behavioural interventions has frequently been criticized for lacking the guidance of a systematic and well-delineated framework, leading to an increased chance of having flaws that cause research waste.^63,64^ Following the IDEAS framework, the development of MoodMover was primarily informed by the results of a comprehensive, systematic review for identifying intervention needs^17^ and a theoretical paper evaluating the use of the M-PAC framework among individuals with poor mental health.^35^ The need for an app specifically designed to increase PA among people with depression was further highlighted by our participants during the interviews. The content of MoodMover was adapted from a web-based M-PAC intervention for PA^47^ and enhanced through a curated selection of behavioural strategies based on prior literature, as well as consultations with experts in the fields of depression and PA. In addition, the research team improved the content design (e.g., assure confidentiality and add institutional logo to images to improve credibility) by considering the barriers and facilitators that individuals with depression face when engaging with mHealth technologies to improve the usability, acceptability, and adoption of MoodMover.^50,51^

The collection of both qualitative and quantitative data from end-users provided a comprehensive understanding of the usability of MoodMover. Overall, the MAUQ results indicated good usability. Qualitative feedback received from think-aloud processes and semi-structured interviews revealed deeper insights into all aspects of the program. Most identified usability issues were minor and considerable positive feedback was received. Especially, all participants expressed their interests in using an updated version of MoodMover in the future, which adds support to the usability and acceptability of the MoodMover program. The simplicity of the Pathverse platform and the involvement of a Pathverse programmer facilitated rapid prototyping throughout the design phases. This enabled ongoing modifications to MoodMover, allowing for highly iterative development with continuous evaluation by subsequent participants. One potential concern that remained was that some users did not readily recall early instructions after navigating through the different lessons. This issue could be addressed by upgrading the app to allow participants to resume unfinished lessons, thereby enabling them to use the tools while reading the instructions, or by conducting a short orientation session to review the app and its features. Considering time and resources, a 15-min virtual orientation session will be integrated into the program begins in the planned feasibility study.

Features such as gamification with financial incentives, exercise logging with mood tracking, and resources including YouTube workout videos selected by researchers were particularly favoured by participants. In particular, financial incentives have consistently been shown to increase PA participation in the short term, although the effects on sustained PA in adults have yielded mixed results.^65–67^ There is no consensus on whether the use of incentives or rewards inhibits intrinsic motivation, which may impact the maintenance of PA behaviour after the removal of incentives.^65,68^ To our knowledge, no review has evaluated the use of financial incentives in PA behaviour change programs among individuals with depression. Given these, while directly incentivizing the desired outcomes, such as step counts or meeting step goals, were more likely to increase step counts post-intervention,^67^ MoodMover instead incentivizes lesson completion. This approach aims to enhance participant engagement with the content, which can potentially lead to increased PA participation and mitigate the impact of removing incentives.^69^ Additionally, the integration of points-based gamification feature may potentially enhance this effect.^70^

Exercise logging along with a mood self-tracking component holds promise in increasing PA by enhancing awareness of the positive correlation between PA and improved mood.^71^ The favourable view of this feature in our study is consistent with previous evidence; mood tracking tools are generally well accepted among individuals with mental illnesses.^71^ In addition, Torous et al.^72^ found an average attrition rate of 26.2%, rising to 47.8% when adjusted for publication bias, among mental health apps for depression, with lower rates observed in those with mood tracking (18.4%). The incorporation of mood tracking is likely to increase adherence and engagement of MoodMover.

### Limitations

Although the sample size in our study was deemed sufficient for usability testing^73–76^ and composed of diverse physically inactive participants across different genders, a wide range of age groups, and varying severities of depressive symptoms, ongoing modifications to MoodMover may warrant a larger sample size. However, we ensured that any modifications were reviewed by at least three subsequent participants. In addition, recruiting participants from a single research website and limiting participation to individuals who owned a smartphone potentially introduced selection bias, reducing the representativeness of the sample. This approach may have overlooked the needs of marginalized groups, potentially contributing to existing mental health inequities from the outset of the research. This limitation should be carefully considered in future studies, especially in large-scale trials assessing the effectiveness and implementation of MoodMover. For instance, future studies could consider providing mobile devices on loan to participants who do not own a smartphone. Moreover, the limited sample size hindered the ability to examine the determinants of usability (e.g., gender, age, and severity of depression), which should be further investigated in a larger-scale trial. Furthermore, the interviews were conducted over Zoom to eliminate the geographic barriers facing potential participants and mimic adoption in the real world. While the entire meeting was recorded as a video file, participants had the option to turn off their cameras, making the interpretation of facial expressions impossible. Moreover, not all tasks were assigned to all participants as planned to reduce respondent burden, which may have prevented the identification of additional usability issues. In particular, this led to a less comprehensive understanding of the perceived usability of the ‘goals (action planning)’ and ‘social community forum’ features. In addition, the tested prototypes did not contain the actual podcasts. Participants were informed that podcasts would be used to deliver certain content; therefore, we were unable to obtain feedback on usability issues related to the podcasts. Moreover, most participants had the opportunity to scan through only two or three lessons during the meeting, which limited their ability to provide in-depth feedback on the content within MoodMover. However, since the content was adapted from a previous web-based intervention that had been reviewed by end-users, and the refined content was reviewed by professionals in our multidisciplinary team, we believe this limitation will not unduly impact the usability, acceptability, and potential efficacy of MoodMover.

## Conclusions

This study describes the rigorous development of the first app-based, M-PAC theory-guided intervention aimed at PA promotion designed for people with depression following the IDEAS framework. The comprehensive usability testing indicated good usability of the tested MoodMover prototypes and enabled a user-centred approach to refine the app to better align with end-users' preferences and needs. At this point, the research team reached a consensus to proceed to the next stage of the IDEAS framework – assess – to evaluate the feasibility and preliminary efficacy of the refined intervention package of MoodMover.

## Supplemental Material

sj-docx-1-dhj-10.1177_20552076251317756 - Supplemental material for MoodMover: Development and usability testing of an mHealth physical activity intervention for depression Supplemental material, sj-docx-1-dhj-10.1177_20552076251317756 for MoodMover: Development and usability testing of an mHealth physical activity intervention for depression by Yiling Tang, Madelaine Gierc, Henry La, Juehee Kim, Sam Liu, Raymond W Lam, Eli Puterman and Guy Faulkner in DIGITAL HEALTH

sj-docx-2-dhj-10.1177_20552076251317756 - Supplemental material for MoodMover: Development and usability testing of an mHealth physical activity intervention for depression Supplemental material, sj-docx-2-dhj-10.1177_20552076251317756 for MoodMover: Development and usability testing of an mHealth physical activity intervention for depression by Yiling Tang, Madelaine Gierc, Henry La, Juehee Kim, Sam Liu, Raymond W Lam, Eli Puterman and Guy Faulkner in DIGITAL HEALTH

sj-docx-3-dhj-10.1177_20552076251317756 - Supplemental material for MoodMover: Development and usability testing of an mHealth physical activity intervention for depression Supplemental material, sj-docx-3-dhj-10.1177_20552076251317756 for MoodMover: Development and usability testing of an mHealth physical activity intervention for depression by Yiling Tang, Madelaine Gierc, Henry La, Juehee Kim, Sam Liu, Raymond W Lam, Eli Puterman and Guy Faulkner in DIGITAL HEALTH

sj-docx-4-dhj-10.1177_20552076251317756 - Supplemental material for MoodMover: Development and usability testing of an mHealth physical activity intervention for depression Supplemental material, sj-docx-4-dhj-10.1177_20552076251317756 for MoodMover: Development and usability testing of an mHealth physical activity intervention for depression by Yiling Tang, Madelaine Gierc, Henry La, Juehee Kim, Sam Liu, Raymond W Lam, Eli Puterman and Guy Faulkner in DIGITAL HEALTH

sj-docx-5-dhj-10.1177_20552076251317756 - Supplemental material for MoodMover: Development and usability testing of an mHealth physical activity intervention for depression Supplemental material, sj-docx-5-dhj-10.1177_20552076251317756 for MoodMover: Development and usability testing of an mHealth physical activity intervention for depression by Yiling Tang, Madelaine Gierc, Henry La, Juehee Kim, Sam Liu, Raymond W Lam, Eli Puterman and Guy Faulkner in DIGITAL HEALTH

sj-docx-6-dhj-10.1177_20552076251317756 - Supplemental material for MoodMover: Development and usability testing of an mHealth physical activity intervention for depression Supplemental material, sj-docx-6-dhj-10.1177_20552076251317756 for MoodMover: Development and usability testing of an mHealth physical activity intervention for depression by Yiling Tang, Madelaine Gierc, Henry La, Juehee Kim, Sam Liu, Raymond W Lam, Eli Puterman and Guy Faulkner in DIGITAL HEALTH

sj-docx-7-dhj-10.1177_20552076251317756 - Supplemental material for MoodMover: Development and usability testing of an mHealth physical activity intervention for depression Supplemental material, sj-docx-7-dhj-10.1177_20552076251317756 for MoodMover: Development and usability testing of an mHealth physical activity intervention for depression by Yiling Tang, Madelaine Gierc, Henry La, Juehee Kim, Sam Liu, Raymond W Lam, Eli Puterman and Guy Faulkner in DIGITAL HEALTH
