# Supplementary material for: MoodMover: Development and usability testing of an mHealth physical activity intervention for depression
Source: Digit Health. 2025 Feb 3;11:20552076251317756. doi: 10.1177/20552076251317756 (PMC11792034; doi:10.1177/20552076251317756)
Supplement: sj-docx-1-dhj-10.1177_20552076251317756 - Supplemental material for MoodMover: Development and usability testing of an mHealth physical activity intervention for depression [file sj-docx-1-dhj-10.1177_20552076251317756.docx]

**Appendix 1.***Outline of the content of MoodMover and the corresponding M-PAC constructs and behaviour change techniques*

| **Modules** | **Topics covered** | **M-PAC construct targeted** | **Behaviour change technique used** |
| --- | --- | --- | --- |
| **1) Feeling better through daily activity** | - Exercise as a treatment option for depression - Mood and physical activity interaction - Physical benefits of exercise - Goal-setting | Affective attitudes, Instrumental attitude;  Behavioural regulation | Information about health consequences (5.1)*  Information about emotional consequences (5.6)*  Behavioural practice/rehearsal (8.1)*  Goal setting (behaviour) (1.1)*  Self-monitoring of behaviour (2.3)*  Credible source (9.1)* |
| **Complementary module 1** | - Reward progress - Overcome low mood | Affective attitudes;  Behavioural regulation | Self-incentive (10.7)*  Self-reward (10.9)*  Problem solving (1.2)*  Reduce negative emotions (11.2)* |
| **2) Making physical activity enjoyable** | - Introducing affect - The importance of enjoying physical activity - Strategies and activities to increase the enjoyment of physical activity | Affective attitudes;  Behavioural regulation | Information about emotional consequences (5.6)*  Behavioural practice/rehearsal (8.1)*  Self-monitoring of behaviour (2.3)* |
| **Complementary module 2** | - Motivation to exercise - Overcome bad weather - Workout videos | Behavioural regulation | Problem solving (1.2)* |
| **3) Building your self-confidence** | - Self-efficacy - How to increase self-efficacy - Exercise experiences shared by peers | Perceived capability  Behavioural regulation | Goal setting (behaviour) (1.1)*  Self-monitoring of behaviour (2.3)*  Information about others’ approval (6.3)*  Behavioural practice/rehearsal (8.1)* |
| **Complementary module 3** | - More exercise experiences | Instrumental attitude | Information about others’ approval (6.3)* |
| **4) Building your physical activity opportunity** | - The influence of environment on behaviour - How to build environment for physical activity - Brainstorm physical activity opportunities - Grab and go activities | Perceived opportunity | Goal setting (behaviour) (1.1)*  Self-monitoring of behaviour (2.3)*  Prompts/cues (7.1)*  Behavioural practice/rehearsal (8.1)*  Restructuring the physical environment (12.1)*  Adding objects to the environment (12.5)* |
| **Complementary module 4** | - Overcome fatigue - Except physical sensations - Mindfulness | Behavioural regulation | Problem solving (1.2)*  Instruction on how to perform a behaviour (4.1)* |
| **5) Developing self-regulatory skills** | - Action planning - Coping planning | Behavioural regulation | Goal setting of behaviour (1.1)*  Problem solving (1.2)*  Action planning (1.4)*  Self-monitoring of behaviour (2.3)*  Behavioural practice/rehearsal (8.1)* |
| **Complementary module 5** | - Protected time | Behavioural regulation | Action planning (1.4)*  Behavioural practice/rehearsal (8.1)* |
| **6) Drawing on social support** | - Introducing social support - Build your social support - Thinking strategies for exercise - Positive self-talk | Perceived opportunity  Behavioural regulation | Goal setting (behaviour) (1.1)*  Self-monitoring of behaviour (2.3)*  Social support (practical) (3.2)*  Social support (emotional) (3.3)* Behavioural practice/rehearsal (8.1)*  Self-talk (15.4)* |
| **Complementary module 6** | - Positive reflective and self-talk | Behavioural regulation | Self-talk (15.4)* |
| **7) Forming an exercise habit** | - Introducing habit - Relating habit to physical activity - How to form a habit (repetition, scripts, environmental cues) | Habit | Goal setting (behaviour) (1.1)*  Self-monitoring of behaviour (2.3)*  Behavioural practice/rehearsal (8.1)*  Habit formation (8.3)* |
| **Complementary module 7** | - Hating some exercise sessions is OK | Behavioural regulation | Self-talk (15.4)* |
| **8) Building your exercise identity** | - Introducing WHO guidelines - Introducing exercise identity - Ways to increase exercise identity (commitment, enjoyment, social comparison, passion) | Identity | Goal setting (behaviour) (1.1)*  Self-monitoring of behaviour (2.3)*  Behavioural practice/rehearsal (8.1)*  Incompatible beliefs (13.3)*  Valued self-identity (13.4)* |
| **Complementary module 8** | - A surprise, bonus lesson with encouraging messages | Affective attitudes | Social reward (10.4)* |

*Note.* M-PAC = Multi-Process Action Control; WHO = World Health Organization.

*The numbers in the brackets refers to the behaviour change techniques in the “BCT taxonomy v1” by Michie et al (36)
