# Supplementary material for: MoodMover: Development and usability testing of an mHealth physical activity intervention for depression
Source: Digit Health. 2025 Feb 3;11:20552076251317756. doi: 10.1177/20552076251317756 (PMC11792034; doi:10.1177/20552076251317756)
Supplement: sj-docx-4-dhj-10.1177_20552076251317756 - Supplemental material for MoodMover: Development and usability testing of an mHealth physical activity intervention for depression [file sj-docx-4-dhj-10.1177_20552076251317756.docx]

**Appendix 4.** *Demographic survey.*

1. What is your age?
2. Please indicate your gender.

- Woman
- Man
- Non-binary person
- Prefer not to Answer

1. The Canadian Census identifies the following categories in its Census of the Population. Please indicate how you self-identify. This self-identification is not intended as an indication of one’s place of origin, citizenship, language or culture and recognizes that there are differences both between and among subgroups of persons of colour. If you are of mixed-descent, please indicate this by selecting all that apply, rather than using the “other” line unless parts of your self-identification do not appear in this list.

- Indigenous peoples of Canada
- Indigenous (outside of Canada)
- Arab
- Black
- Chinese (including Mainland China, Hong Kong, Macau and Taiwan)
- Filipino
- Japanese
- Korean
- Latin, Central, or South American (e.g., Brazilian, Chilean, Colombian, Mexican)
- South Asian (e.g., Indian, Pakistani, Sri Lankan, etc.)
- Southeast Asian (e.g., Cambodian, Indonesian, Laotian Vietnamese, etc.)
- West Asian (e.g., Afghan, Iranian, Syrian, etc.)
- White
- Other
- Prefer not to answer

1. Please indicate your ancestry here (if “Other” is selected to Q3).
2. What is the highest degree or level of education you have completed?

- Some high school
- High school
- Certificate or diploma
- Bachelor’s degree
- Master’s degree
- PhD
- Professional degree
- Prefer not to answer

1. What is your current employment status?

- Employed – part time (including self-employed)
- Employed – full time (including self-employed
- Stay-at-home parent
- Homemaker
- On maternity/parental leave
- On medical or disability
- Retired
- Unemployed
- Other
- Prefer not to answer

1. Please indicate your current employment status here (if “Other” is selected to Q6).
2. What is your before-tax household income?

- $0 - $19,999
- $20,000 - $39,999
- $40,000 - $59,999
- $60,000 - $79,999
- $80,000 - $99,999
- $100,000 - $119,999
- $120,000 - $139,999
- $140,000 or greater
- Prefer not to answer
