# Supplementary material for: MoodMover: Development and usability testing of an mHealth physical activity intervention for depression
Source: Digit Health. 2025 Feb 3;11:20552076251317756. doi: 10.1177/20552076251317756 (PMC11792034; doi:10.1177/20552076251317756)
Supplement: sj-docx-5-dhj-10.1177_20552076251317756 - Supplemental material for MoodMover: Development and usability testing of an mHealth physical activity intervention for depression [file sj-docx-5-dhj-10.1177_20552076251317756.docx]

**Appendix 5.** *Semi-structured interview questions.*

Q1: Do you use, or have you used, any mental health/physical activity apps/wearable devices? Why/why not?

Q2: What did you like best about the MoodMover app?

Q3: What did you like least about the app?

Q4: How easy was it to navigate or find your way around the app?

Q5: What did you think about the overall look of the app?

Q6: What did you think about the information provided on the app?

Q7: Is there anything you think the app might be missing?

Q8: Would you be interested in using the upgraded MoodMover in the future to increase your physical activity and reduce depressive symptoms? Why/why not?
