# Supplementary material for: MoodMover: Development and usability testing of an mHealth physical activity intervention for depression
Source: Digit Health. 2025 Feb 3;11:20552076251317756. doi: 10.1177/20552076251317756 (PMC11792034; doi:10.1177/20552076251317756)
Supplement: sj-docx-6-dhj-10.1177_20552076251317756 - Supplemental material for MoodMover: Development and usability testing of an mHealth physical activity intervention for depression [file sj-docx-6-dhj-10.1177_20552076251317756.docx]

**Appendix 6.** *Adapted MAUQ – Patient version for standalone apps*

(Responses on a 1 to 7, Strongly Disagree to Strongly Agree, scale)

**Ease of use**

S1. The app was easy to use.

S2. It was easy for me to learn to use the app.

S3. The navigation was consistent when moving between screens.

S4. The interface of the app allowed me to use all the functions (such as setting a step goal, logging a physical activity session, receiving notifications) offered by the app.

**Interface and satisfaction**

S5. Whenever I made a mistake using the app, I could recover easily and quickly.

S6. I like the interface of the app.

S7. The information in the app was well organized, so I could easily find the information I needed.

S8. The app adequately acknowledged and provided information to let me know the progress of my action.

S9. I feel comfortable using this app in social settings.

S10. The amount of time involved in using this app has been fitting for me.

S11. I would use this app again.

S12. Overall, I am satisfied with this app.

**Usefulness**

S13. The app would be useful for my mental health and well-being.
