# Supplementary material for: MoodMover: Development and usability testing of an mHealth physical activity intervention for depression
Source: Digit Health. 2025 Feb 3;11:20552076251317756. doi: 10.1177/20552076251317756 (PMC11792034; doi:10.1177/20552076251317756)
Supplement: sj-docx-7-dhj-10.1177_20552076251317756 - Supplemental material for MoodMover: Development and usability testing of an mHealth physical activity intervention for depression [file sj-docx-7-dhj-10.1177_20552076251317756.docx]

**Appendix 7.** *Participant characteristics (N=9).*

| Characteristics | n |
| --- | --- |
| Gender |  |
| Woman | 5 |
| Man | 3 |
| Non-binary | 1 |
| Age, mean (SD) | 38.4 (10.5) |
| Age group (years) |  |
| 18-30 | 2 |
| 30-40 | 4 |
| 40-50 | 1 |
| 50-60 | 2 |
| Ethnicity |  |
| White | 5 |
| White and Indigenous | 1 |
| Chinese | 1 |
| South Asian | 1 |
| Latin, Central, or South American | 1 |
| Education Level |  |
| High school | 1 |
| Certificate or diploma | 1 |
| Bachelor | 4 |
| Master | 3 |
| Employment statue |  |
| Employed (full-time) | 3 |
| Employed (part-time) | 3 |
| On medical or disability | 3 |
| Income level |  |
| $20,000 - $39,999 | 1 |
| $40,000 - $59,999 | 1 |
| $60,000 - $79,999 | 1 |
| $80,000 - $99,999 | 2 |
| $100,000 - $119,999 | 1 |
| $120,00 - $139,999 | 2 |
| $140,000 or greater | 1 |
| PHQ-9, mean (SD) | 14.1 (6.47) |
| Mild: 5-9 | 3 |
| Moderate: 10-20 | 4 |
| Severe: 20-27 | 2 |
| Physical activity, n of days/week with at least 30 mins of MVPA |  |
| 0 | 3 |
| 1 | 4 |
| 2 | 2 |

*Note*. MVPA = Moderate-to-vigorous physical activity; PHQ-9 = Patient Health Questionnaire – 9 items.
